# Catheter-associated urinary tract infections in adult intensive care units at a selected tertiary hospital, Addis Ababa, Ethiopia

**DOI:** 10.1371/journal.pone.0265102

**Published:** 2022-03-22

**Authors:** Hiwot Bizuayehu, Adane Bitew, Abera Abdeta, Semira Ebrahim

**Affiliations:** 1 Department of Microbiology, Addis Ababa Burn, Emergency and Trauma Hospital, Addis Ababa, Ethiopia; 2 Department of Medical Laboratory Sciences, College of Health Sciences, Addis Ababa University, Addis Ababa, Ethiopia; 3 National Clinical Bacteriology and Mycology Reference Laboratory, Ethiopian public health institute, Addis Ababa, Ethiopia; Tribhuvan University, NEPAL

## Abstract

**Background:**

Catheter-associated urinary tract infection is the leading cause of hospital-acquired infections. They remain the second most common healthcare-associated infection in critically sick patients.

**Objective:**

To determine the prevalence of catheter-associated urinary tract infection, the spectrum of etiological agents, antibiotic sensitivity profile of bacterial pathogens among adult patients admitted to intensive care units.

**Materials and methods:**

Patients admitted to the intensive care unit of hospitals in Addis Ababa who were on urethral indwelling catheters for >48 hours from October 2020 to September 2021 were included in the study. Urine specimens were aseptically collected and processed as per standard protocols. Microorganisms were isolated, identified, and subjected to antibiotic susceptibility testing.

**Results:**

In all 220 pateints included in the study development of significant bacteriuria/candiduria was not affected by sex, age, and prior antibiotic therapy. However, the length of stay in the intensive care unit was significantly associated with bacteriuria /candiduria (P-value < 0.001). The overall prevalence of bacteriuria/candiduria was 51.4% among which 21.0%, 19.1%, and 11.4% were bacteriuria, candiduria, and polymicrobial infections, respectively. About 138 organisms were recovered of which 79 (57.25%) were bacteria and 59 (42.75%) were yeast isolates. A*cinetobacter* species, *Pseudomonas* species, *Klebsiella species E*. *coli*, *and Enterococcus species were the* dominant bacterial isolates. *Candida*. *albicans*, *Candida*. *krusei* and *Candida*. *tropicalis* were the commonest yeasts. Many gram-negative bacterial isolates were resistant to ceftriaxone 36(94.7%), ampicillin 21(91.3%) followed by cefotaxime34(89.5%), amikacin (16.0%), nitrofurantoin (17.4%), meropenem (20.0%) and imipenem (20.0%). Out of 79 bacterial pathogens, 52(65.8%) were multiple antibiotic resistant of which 37(71.0%) were Gram-negative bacteria and 15(29%%) were Gram-positive bacteria. About 13(86.7%) isolates of *Acinetobacter*, all isolates of *Klebsiella* species (100%) and *E*. *coli* (100%) were multiple antibiotic-resistant. Out Of 18,10(55.56%), isolates of *Enterococcus* were multiple antibiotic-resistant.

**Conclusions:**

A very high prevalence of bacteriuria/ candiduria was demonstrated in this study. This warrants the establishment of multidimensional infection control approach on catheter associated urinary tract infection in ICU. In addition to high prevalence of candiduria, recovery of non-albicans candida species almost in equal proportion with candida albicans in the present study was an important finding as non-albicans candida species distinct to C. albicans are intrinsically resistant to the commonly used azole antifungal drugs in Ethiopia. The prevalence of rate MDR bacteria in our ICU particularly of *E*. *coli*, *Klebsiella* spp, *Pseudomonas and Acetobacter* spp was very high. In order to combat this problem, proper antibiotic policies should be formulated.

## Introduction

Health-care-associated infections (HCAIs) are among the most serious health problems as they are associated with high morbidity and mortality rates, prolonged hospital stay, and increased costs of therapy [1, 2]. They are primarily classified as ward or intensive care unit (ICU) acquired infections. Intensive care units present a specific setting in which HCAIs are acquired at a higher rate and exhibit higher mortality [3]. The prevalence of ICU-acquired infections (ICUAI) is significantly higher in developing countries than in developed countries [4, 5]. According to the World Health Organization [5] factsheet, the frequency of ICUAI in developing countries is 2–3 times higher than in developed countries.

Healthcare-associated urinary tract infection (HCAUTI) is one of the six major HCAIs [6] and also one of the most common health problems in patients admitted to intensive care units [7–10]. Salgado et al [10] and Foxman [11] reported that this condition accounts for up to 40% of the 2 million HCAIs each year.

Catheter-associated urinary tract infection (CAUTI) has been described as one of the most common device-associated HCAIs developed frequently by patients hospitalized in the ICU. Closely 75% of patients admitted to hospitals undergo urinary catheterization in the course of their hospital stay, making prevention and control of CAUTI difficult [12]. In the intensive care unit, 95% of UTIs are CAUTIs and despite efforts to reduce CAUTI incidence, they remain the second most important HCAIs in critically ill patients [12, 13]. Left undiagnosed and untreated, CAUTI increases the risk for complications and ultimately decreases the efficiency of health care delivery. Therefore, timely intervention is needed to prevent complications such as cystitis, acute or chronic pyelonephritis, bacteremia, and urosepsis [14].

Among several etiological agents of ICUAUTI, Gram-positive bacteria such as *Staphylococcus species*, *Enterococcus faecalis and* Gram-negative bacteria *such Escherichia coli*, *Pseudomonas specis*, *Proteus mirabilis*, *Klebsiella species*, *Enterobacter species*, and yeasts such as *Candida species* are the most frequently reported [15]. Drug resistance development in urinary tract infection causing pathogens is a growing problem and the rate of development is much faster in the ICUs compared with other hospital wards and outpatient clinics demonstrating that hospitalization in ICUs may be an independent risk factor for acquiring infection by multiantibiotic-resistant strains [16].

Despite a high global burden of CAUTI, there has been no study conducted on UTI in critically ill patients in Ethiopia. To this effect, the objectives of the current study are to determine the prevalence of CAUTI, the spectrum of etiological agents, antibiotic resistance profile of bacterial pathogens, and major risk factors associated with CAUTI.

### Operational definitions

1. Individual bacteriuria and candiduria were defined by the presence of a positive urine culture collected in ICU, with no more than two pathogens, not isolated previously, and at ≥ 10^5^ colony-forming units (cfu)/ml [17]
2. Hospital-acquired or healthcare-associated bacteriuria/candiduria were defined as positive urine cultures occurring after 48 h of hospital admission in patients admitted from home.
3. Positive urine cultures were classified as ward-acquired bacteriuria/candiduria when collected within the first 48 h of ICU stay and ICU-acquired bacteriuria/ candiduria when collected after 48 h of ICU stay.
4. Multi-drug resistance (MDR): non-susceptibility to at least one agent in three or more antimicrobial categories.
5. Significant bacteriuria/ candiduria is defined as a urine sample containing more than 10^5^ colony-forming units/ml of urine in pure culture using a standard calibrated bacteriological loop.

## Materials and methods

### Study area, design, and period

This hospital-based cross-sectional study was conducted on 220 catheters indwelled adult ICU patients in three tertiary care hospitals, i.e., St Paul’s Millennium Medical College with six beds, Ras Desta Damtew Memorial Hospital with six beds, and Aabet Hospital with twelve beds from October 2020 to September 2021, Addis Ababa, Ethiopia. The urine specimens were brought to the Ethiopian Public Health Institutes National clinical bacteriology and mycology reference laboratory for culture and antimicrobial susceptibility.

### Inclusion criteria

Patients ≥ 16 years of age, having at least two calendar days of urinary catheterization after being admitted to the intensive care unit, and giving informed written consent were included in the study. Patients with positive urine culture before catheterization and less than 2 calendar days of urinary catheterization were excluded from the study.

### Demographic characteristics of patients

Demographic information such as age, sex, clinical diagnosis, reasons for hospitalization and sources of admission to the ICU, and history of systemic antibiotic treatment were recorded.

#### Clinical sample collection and Inoculation

Sterile urethral indwelling catheters were aseptically applied to the patients by trained physicians in a way that minimizes the risk of introducing microorganisms to the bladder following the hospital guidelines or standard operating procedures of the hospitals.

About 10 ml of urine sample was aspirated from an indwelling catheter near the urethral site under aseptic precautions from all patients. Urine specimens were collected from each patient with a sterile urine container by trained nurses or physicians. Immediately after collection, the samples were brought to the microbiology laboratory of the Ethiopian Public Health Institute (EPHI) for further processing. Part of the sample was inoculated onto MacConkey agar plate (Oxide, Basingstoke, and Hampshire, England) and sheep blood agar medium using a calibrated loop with a capacity of 1 μl in a safety cabinet. The remaining sample was inoculated onto Brain Heart Infusion agar supplemented with chloramphenicol (100μgml^-1^) and gentamycin (50 μgml^-1^) (Oxide, Basingstoke, and Hampshire, England).

#### Incubation

All inoculated plates were incubated at 37°C for 18–48 hours aerobically and inspected for the growth of bacteria and/or yeasts. Colony counts yielding bacterial or yeast growth of ≥10^4^ and ≥10^5^/ ml for single uropathogen and two uropathogens respectively were regarded as significant for bacteriuria or candiduria. Urine samples that yielded three or more species were not considered for further investigation.

#### Bacterial identification

Pure culture isolates of the bacterial pathogen were preliminarily characterized by colony morphology and Gram stain. Bacterial isolates were identified down to the species level by using standard biochemical tests.

#### Yeast identification

Yeasts were identified by employing conventional routine diagnostic methods such as Gram stain, Germ tube test, carbohydrate fermentation, and assimilation test, and by employing chromogenic medium (CHROMagar Candida medium, bioM´erieux, France) as per the instruction of the manufacturer.

#### Antimicrobial susceptibility testing

Antimicrobial susceptibility test was carried out by standardized Kirby Bauer disc diffusion method as per Clinical Laboratory Standards Institute (CLSI) M100 guidelines [18] on Muller Hinton agar (Oxoid, Basingstoke, England). Bacterial suspension of each isolate was prepared in 0.5 ml of nutrient broth medium and the turbidity was adjusted to match that of 0.5 McFarland standards. A sterile swab was dipped into the suspension and then the swab was applied to the center of the Muller Hinton agar plate and evenly spread on the medium. Antibiotic discs were placed onto Muller Hinton agar seeded with each isolate and were incubated for 24 hours at 35–37°C. Inhibition zone (IZ) diameter was measured using a caliper and interpreted according to the Clinical and Laboratory Standards Institute (CLSI) guideline [18] as susceptible (S), intermediate (I), or resistant (R). *Staphylococcus* aureus *(*ATCC 25923), *Escherichia coli* (ATCC 25922), and *Pseudomonas aeruginosa* (ATCC 27853) were used as quality control strains.

#### Classes of antibiotics used

Antibiotic disc used in the present study were: Aminoglycosides (Gentamicin 10μg, Amikacin 10μg, Tobramycin 10μg), Cephalosporins (Cefotaxime 30μg, Ceftazidime 30μg Ceftriaxone 30μg) Nitrofurantoin (Nitrofurantoin 300μg),tetracyclines (tetracycline 30μg),Folate Pathway Inhibitors (trimethoprim- sulfamethoxazole 25μg), Penicillins (oxacillin 30μg, penicillin10μg, Ampicillin 10μg), Glycopeptides (vancomycin 30μg), Carbapenems (Imipenem 10μg, Meropenem 10μ), Fluoroquinolones(ciprofloxacin 5μg). All the antibiotics used in the study were the products of Oxoid, Basingstoke, and Hampshire, England

### Statistical analysis

All data from the investigation were coded, double entered, and analyzed using Statistical Package for Social Sciences (SPSS) software version 23. Descriptive statistics and logistical regressions were used to estimate the crude ratio with a 95% confidence interval to the different variables. P-value < 0.05 was considered significant.

### Ethical approval and consent to participate

Ethical clearance was obtained from the Department Research and Ethical Review Committee of the Department of Medical Laboratory Sciences, College of Health Sciences, Addis Ababa University (MLS/215/19). Permission was obtained from the Ethiopian Public Health Institute. The written informed consent was obtained from patients, their names and other personal identifiers were encrypted.

## Results

### Patient characteristics

As shown in Table 1, two hundred and twenty (220) patients that met the selection criteria were recruited into the study. The number of male study participants (144; 65.5%) outnumbered that of female study participants (76; 34.5%) where the male-female ratio was 1.9: 1. The number of study subjects enrolled per age group was variable in which patients with an age group of 25–64 were the highest (136;61.8%. The study subjects were urban dominated, where the urban-rural ratio being 2.4:1. Most of the patients were admitted into the ICU from the emergency department and surgical wards. Similarly, diseases of the circulatory system, the respiratory system, and injury were the major reasons for hospitalization. About 115 patients stayed in the ICU for less than 5 days while 105 stayed four about six or more days in the ICU where the duration of stay in the ICU was between 2 and 50 days, with a median of 5 ± 8 days. International Statistical Classification of Diseases and Related Health Problems 10th Revision (ICD-10)-WHO Version for; 2016 [19] was used for the classification of reasons of hospitalization.

**Table 1:** Patients demographic data and clinical history.

Variables		Number	%
Gender	Male	144	65.5
	Female	76	34.5
Age	16–24	49	22.3
	25–64	136	61.8
	≥ 65	35	15.9
Residence	Urban	156	70.9
	Rural	64	29.1
Antibiotic treatment	Yes	192	87.3
	No	28	12.7
Length of the day in the ICU	≥6	105	47.7
	≤5	115	52.3
Source of admission to the ICU	Emergency	137	62.3
	Medical	14	6.4
	Surgical	38	17.3
	Gynecology and Obstetrics	5	2.3
	Community	15	6.8
	Operation room	11	5
Cause of hospitalization (18)	Certain infectious and parasitic diseases	29	13.2
	Endocrine, nutritional and metabolic diseases	23	10.5
	Injury, Poison, External causes	34	17
	Diseases of the circulatory system	50	22.7
	Diseases of the Respiratory system	40	18.2
	Diseases of the Digestive system	12	5.5
	Diseases of the genitourinary system	9	4.1
	Diseases of the blood and blood-forming organs	5	2.3
	Cancer, nervous system, other diseases	18	8.2

### Prevalence of bacteriuria/ candiduria

Out of two hundred and twenty (220), urine samples collected bacteriuria/ candiduria were observed in 113 samples giving an overall prevalence of 51.4% (113/220). The prevalence of bacteriuria and candiduria was 21.0% (46/2220), and 19.0% (42/220), respectively. The remaining 11.4% (25/220) were polymicrobial infections among which five samples yielded two different yeast pathogens each, 13 samples yielded two different bacterial pathogens, and the remaining seven samples yielded bacterial and yeast pathogen concurrently (Table 2).

**Table 2:** The prevalence of catheter-associated bacteriuria/candiduria and the spectrum of etiologic agents.

Micro-organism	Pure	Mixed	Total
*Acinetobacter spp*	9	6	15
*Citrobacter spp*.	0	4	4
*E*. *coli*	5	3	8
*Enterobacter spp*.	0	2	2
*Klebsiella oxytoca*	2	-	2
*Klebsiella pneumonia*	1	1	2
*Klebsiella ozaenae*	2	3	5
*Pseudomonas spp*.	8	4	12
*Enterococcus spp*.	11	7	18
*S*. *epidermidis*	8	3	11
**Total bacterial isolates**	**46**	**33**	**79**
*Candida albicans*	21	8	29
*Candida krusei*	15	3	18
*Candida tropicalis*	4	6	10
*Cryptococcus neoformans*	2	-	2
**Total yeast isolates**	**42**	**17**	**59**
**Mixed cultures**			
*Acinetobacter spp and Enterococcus spp*.		1	1
*Acinetobacter spp and S*. *epidermidis*		1	1
*Acinetobacter spp and Citrobacter spp*.		1	1
*Citrobacter spp*. *and Pseudomonas spp*.		1	1
*Citrobacter spp*. *and Enterobacter spp*.		2	2
*E*. *coli and Acinetobacter spp*		1	1
*E*. *coli and Pseudomonas spp*.		1	1
*Klebsiella ozaenae and Acinetobacter spp*		1	1
*Klebsiella ozaenae and Pseudomonas spp*.		1	1
*Klebsiella pneumonia and Enterococcus spp*.		1	1
*Pseudomonas spp*. *and Enterococcus spp*.		1	1
*S*. *epidermidis and Enterococcus spp*.		1	1
*Candida albicans and Candida krusei*		1	1
*Candida krusei and Candida tropicalis*		2	2
*Candida tropicalis and Candida albicans*		2	2
*Acinetobacter spp and Candida albicans*		1	1
*E*. *coli and Candida albicans*		1	1
*Enterococcus spp*. *and Candida tropicalis*		2	2
*Enterococcus spp*. *and Candida albicans*		1	1
*Klebsiella ozaenae and Candida albicans*		1	1
*S*. *epidermidis and Candida albicans*		1	1
**Total**			**25**

#### Diversity of microbial isolates

A total of 138 bacteria and yeasts were recovered of which 79 (57.25%) were bacterial and 59 (42.75%) were yeast isolates. Of 79 bacterial isolates, 50 (63.3%) were Gram-negative bacteria and 29 (36.7%) were Gram-positive bacteria. Among Gram-negative bacterial isolates, *Acinetobacter* species was the predominant species consisting of 30.0% of bacterial isolates and this was followed by *Pseudomonas* species, (24.0%), *Klebsiella* species (22.0%), and *E*. *coli* (16.0%). Of Gram-positive bacteria, *Enterococcus* species were the dominant one accounting for 62.1% of the total Gram-positive bacterial isolates followed by *S*. *epidermidis* with 37.9%. Among the 59 yeast isolates, the most frequently isolated species was *Candida albicans* accounting for 29 (49.2%) followed by *C*. *krusei*, *and C*. *tropicalis* consisting of 18 (30.5%) and 10 (16.9%) isolates, respectively (Table 2).

### Risk factors

The statistical analysis of the possible risk factor for CAUTI in ICU patients is shown in (Table 3). The prevalence of bacteriuria/candiduria was the highest in the age group of 25–64 years of age and females with 60.5% as compared to males 46.5%. Similarly, UTI was higher in patients admitted from urban than from rural regions. However, the differences in the male to female ratio and age between the bacteriuric/candiduric and non-bacteriuric/candiduric patients were not statistically significant (P value:0.05. and *p* P value:0.92, respectively). The ICU length of stay was significant between bacteriuric/candiduric and non-bacteriuric/candiduric patients ((p = < 0.001).

**Table 3:** Association bacteriuria/candiduria with possible risk factors.

Variable		Frequency	Growth		P value
			Yes	No	
Gender	Female	76	46	30 (39.5)	0.05
	Male	144	67	77 (53.5)	
Age	16–24	49	26	23(46.9)	0.92
	25–64	136	70	66(48.5)	
	> = 65	35	17	18(51.4)	
Residential	Urban	156	83	73(46.8)	0.39
	Rural	64	30	34(53.1)	
Age in years (Mean)	41+	95	51	44(46.3)	0.64
	< = 40	125	62	63(50.4)	
Length in the ICU in days	≥6	105	73	32(30.5)	0.00
	< = 5	115	40	75(65.2)	
Prior antibiotic treatment	Yes	192	97	95(49.5)	0.51
	No	28	16	12(42.9)	
**Total**	** **	**220 (100)**	**113(51.4)**	**107(48.6)**	** **

#### Antimicrobial susceptibility profile of bacterial isolates

The overall antibiotic resistance pattern of Gram-negative bacteria against the thirteen antibiotics evaluated is depicted in Table 4. Gram-negative bacteria were resistant toceftriaxone 36(94.7%), ampicillin 21(91.3%), cefotaxime 34(89.5%), and trimethoprim-sulfamethoxazole 34(81.6%) among the antibiotics tested. Amikacin 8(16.0%), nitrofurantoin 4(17.4%), meropenem 10 (20.0%), and imipenem 10(20.0%) were the most effective antibiotics against Gram-negative bacteria tested. *Acinetobacter* species, the most commonly recovered species were more resistant to Ceftazidime (100%), Ceftriaxone (100%), and trimethoprim-sulfamethoxazole (80.0%). However, these isolates were sensitive toaminoglycosides and carbapenems with a resistance rate ranging from 20–34%. The isolates of *Pseudomonas* species, the next dominant Gram-negative bacteria were less resistant to aminoglycosides and carbapenems with a resistance rate of 16.7%. All isolates of *E*. *coli* were 100% susceptible to aminoglycosides, carbapenems, and nitrofurantoin. *Klebsiella* species were better sensitive against aminoglycosides, carbapenems, and nitrofurantoin with a resistance rate extending from 0–60%. Gram-positive bacteria were relatively less resistant compared to Gram-negative bacteria (Table 5).

**Table 4:** Percentage antimicrobial resistance profile of Gram-negative bacteria against 13 antibiotics.

		Number resistant (Percentage)												
	Isolate (number)	Ampicillin	Ceftazidime	Ceftriaxone	Cefotaxime	Ciprofloxacin	Trimethoprim-sulfamethoxazole	Gentamicin	Tobramycin	Amikacin	Imipenem	Meropenem	Nitrofurantoin	Tetracycline
*Acinetobacter spp(15)*		NT	15(100)	15(100)	13(86.7)	8(53.3)	12(80)	5(33.3)	5(33.3)	3(20)	5(33.3)	5(33.3)	NT	7(46.7)
*K*.*oxytoca (2)*		2(100)	2(100)	2(100)	2(100)	1(50)	2(100)	2(100)	2(100)	0(0)	0(0)	0(0)	0(0)	2(100)
*K*.*pneumoniae (2)*		2(100)	2(100)	2(100)	2(100)	2(100)	2(100)	2(100)	1(100)	1(50)	0(0)	0(0)	1(50)	1(50)
*E*. *coli (8)*		8(100)	3(37.5)	8(100)	8(100)	5(62.5)	8(100)	0(0)	0(0)	0(0)	0(0)	0(0)	0(0)	4(57.1)
*Pseudomonas spp*. *(12)*		NT	10(83.3)	NT	NT	7(58.3)	NT	5(41.7)	5(41.7)	2(16.7)	2(16.7)	2(16.7)	NT	-
*K*.*ozaenae (5)*		5(100)	3(60)	5(100)	5(100)	3(60)	5(100)	3(60)	3(60)	2(40)	3(60)	3(60)	1(20)	5(100)
*Citrobacter spp*. *(4)*		3(75)	1(25)	3(75)	3(75)	1(25)	3(75)	1(25)	1(25)	0(0)	0(0)	0(0)	2(50)	2(50)
*Enterobacter spp*. *(2)*		1(50)	0(0)	1(50)	1(50)	0(0)	2(100)	0(0)	0(0)	0(0)	0(0)	0(0)	0(0)	1(50)
*Total 50*		21(91.3)	36(72.0)	36(94.7)	34(89.5)	27(54.0)	34(89.5)	18(36.0)	17(34.0)	8(16.0)	10(20.0)	10(20.0)	4(17.4)	22(57.9)

NT: Not tested

**Table 5:** Percentage antibiotic resistance profile of Gram-positive bacteria against ten antibiotics.

	Number resistant (Percentage)								
Isolate(number)	Ampicillin	Oxacillin	Penicillin	Ciprofloxacin	trimethoprim-sulfamethoxazole	Gentamycin	Nitrofurantoin	Tetracycline	Vancomycin
*Enterococcus* spp.(18)	13(72.2)	NT	12(66.7)	8(44.4)	NT	NT	4(22.2)	11(61.1)	6(33.3)
*S*. *epidermidis* (11)	NT	9(81.8)	7(63.6)	3(27.3)	7(63.6)	3(27.3)	1(9.1)	2(18.2)	NT
Total 29	-	-	19(65.5)	11(38)	**-**	-	5(17.2)	13(44.8)	**-**

NT = Not tested

The prevalence rate of MDR in Gram-positive and Gram-negative bacteria is shown in Table 6. Out of 79 bacterial pathogens, 52(65.8%) were MDR. Of this 37(71.0%) were Gram-negative bacteria whereas 15(28.2%%) were Gram-positive bacteria. Out of 15 isolates of *Acinetobacter* species, 13 (86.7%) were MDR while all isolates of *Klebsiella* species (100%) and *E*. *coli* (100%) were MDR. The isolates of *Enterococcus* which is the commonest Gram-positive bacterium 10 in 18(55.6%) were MDR (Table 6).

**Table 6:** Prevalence of multi-antibiotic resistance Gram-positive and Gram-negative bacteria.

Bacterial Isolate (n)	Level of Resistance (n, %)									MDR	
	R0	R1	R2	R3	R4	R5	R6	R7	R8	No N (%)	Yes N (%)
*Acinetobacter spp (15)*	0(0)	0(0)	2(13,3)	7(46.7)	4(26.7)	1(6.7)	1(6.7)	--	--	2(13.3)	13(86.7)
*Citrobacter spp (4)*.	0(0)	0 (0)	1(25.0)	0(0)	2(50.0)	1(25.0)	0(0)	0(0)	0(0)	1(25.0)	3(75.0)
*Enterobacter spp (2) *	0(0.0)	0.(0.0)	1(50.)	1(50.0)	0(0.0)	0(0.0)	0(0.0)	0(0.0	0(0.0)	1(50.0)	1(50.0)
*Klebsiella oxytaca (2) *	0(0.0)	0(0.0)	0(0.0)	0(0.0)	0(0.0)	1(50.0)	1(50.0)	0(0.0)	0(0.0)	0(0.0)	2(100.0)
*Klebsiella pneumoniae (2)*	0(0.0)	0(0.0)	0(0.0)	0(0.0)	0(0.0)	0(0.0)	2(100.0)	0(0.0)	0(0.0)	0(0.0)	2(100.0)
*E*. *coli (8) *	0(0.0)	0(0.0)	0(0.0)	2(25.0)	3(37.5)	3(37.5)	0(0.0)	0(0.0)	0(0.0)	0(0.0)	8(100.0)
*Pseudomonas spp (12)*	1(8.3)	2(16.7)	6(50.0)	2(16.7)	1(8.3)	--	--	--	--	9(75.0)	3(25.5)
*Klebsiella ozaenae (5) *	0(0.0)	0(0.0)	0(0.0)	0(0.0)	2(40.0)	0(0.0)	0(0.0	2(40.0)	1(20.0)	0(0.0)	5(100.0)
**Total Gram-negative (50)**	**1(2.0)**	**2(4.0)**	**10(20.)**	**12(24.0)**	**12(24.**	**6(12.0)**	**4.(8.0)**	**2(4.0)**	**1(2.0)**	**13(26.0)**	**37(71.0)**
*Enterococcus spp*. *(18)*	1(5.6)	3(16.7)	4(22.2)	6(33.3)	4(22.2)	0(0.0)	--	--	--	8(44.4)	10(55.6)
*S*. *epidermidis (11) *	1(9.1	3(27.3)	2(18.2)	1(9.1)	4(36.4)	0(0.0)	0(0.0)	--	--	6(54.6)	5(45.4)
**Total Gram-positive (29)**	**2(6.9)**	**6(20.7)**	**6(20.7)**	**7(24.1)**	**8(27.6)**	0(0.0)	0(0.0)	--	-	**14(48.3)**	**15(29)**
**Total**	**3(3.8)**	**8(10.1)**	**16(20.3)**	**19(24.1)**	**20(25.3)**	**6(7.6)**	**4(5.1)**	**2(2.5)**	**1(1.3)**	**27(34.2)**	**52(65.8)**

## Discussion

The overall prevalence rate of catheter-associated bacteriuria/ candiduria in the ICU patients in our study was 51.4%. Our result was comparable with the overall prevalence rate of 49.9% ICU acquired bacteriuria/ candiduria reported in a similar study conducted by Aubron et al [20]. A prevalence rate of 21.0% catheter-associated bacteriuria acquired in the adult ICU in the current study was similar to the prevalence rate of 20% ICU-acquired bacteriuria reported by Vinoth et al [21]. Candidauria is very common in the intensive care unit (ICU) of a hospital. The prevalence rate of adult ICU-acquired catheter-associated candiduria in the current study (19.1%) was within the range of the prevalence rates (19.1%-44.4%) reported by previous similar studies [22, 23]. The absence of an established infection control strategy and proper antibiotic management policies in our ICU settings may explain the overall high prevalence rate of catheter-associated bacteriuria/ candiduria. In the present study, 14.4% of ICU-acquired CAUTI was poly-microbial infections. Our findings contradict those of Simpson et al [24] who found that the prevalence of polymicrobial candiduria was 5–10% and that C. glabrata was the most prevalent isolate in conjunction with C. albicans.

Gender, extremes of age, antibiotic use, length of stay in the ICU, diabetes mellitus, immunosuppressive therapy, and indwelling urinary devices have been identified as risk factors associated with an increase of bacteriuria/candiduria in the ICU patients. However, gender was not a risk factor for catheter-associated bacteriuria/candiduria in our ICU patients, as the male to female ratio depicted that no significant difference between the bacteriuric/ candiduric and non-bacteriuric/candiduric patients. Many studies conducted in general hospitals and some ICUs [25, 26] have shown that device-associated UTIs are more common in women, but this risk factor has not been shown in studies specific to ICU patients [27]. There was also no significant difference in age between individuals who developed an ICU-acquired UTI and those who did not. A similar result was reported in ICU patients by Laupland et al [28]. The lack of association of UTI with sex and age may be described by the predominant age bracket of the recruited patients. The ICU length of stay was statistically associated with the developing ICU-acquired UTI in our catheterized patients and our result was in good agreement with results reported from previous studies [27, 29].

Consistent with the results of numerous studies around the globe [21, 29–32], this study also revealed that Gram-negative bacteria were the main causative agents of bacteriuria. Of 79 bacterial isolates, 50 (63.3%) were Gram-negative bacteria of which isolates A*cinetobacter* spp were the predominant bacterial isolates and this was followed by *Pseudomonas species*, *Klebsiella species*, *and E*. *coli i*n their descending order. Our result in terms of the predominant bacterial isolates was contradictory with many other studies that reported *E*. *coli*, *Klebsiella*, and *Enterobacter* species as predominant bacterial isolates. In most of the studies that have been carried out on UTI till today, the most common organism isolated in UTI is *E*. *coli with* a reduction in the frequency in patients with indwelling catheters [33]. Of Gram-positive bacteria, *Enterococcus* species were the dominant spp accounting for 62.1% of the totalGgram-positive bacterial isolates followed by *S*. *epidermidis*. *Staphylococci and Enterococcus* species as dominant bacteria in urine culture have been reported by many similar studies [31].

Multiple studies showed that within hospital settings at least 10%–15% of hospital-acquired UTIs are caused by *Candida* species and candiduria is especially common ICUs [34–36]. In our study, candiduria represented 44.6% (47/220 including polymicrobial infections) of CAUTIs and *C*. *albicans* was the leading cause of candiduria. Although *C*. *albicans* has been the commonest etiologic agent, non-albicans species were isolated in 50.8% of the urine samples. The emergence of non-albicans species may represent the selection of less susceptible species by antifungal agents as fluconazole in particular. Some Candida strains as *C*. *krusei* are less susceptible to fluconazole than *C*. *albicans* [37]. Similar results about emerging species occurrence have been observed in hospitalized patients [38]. Environmental conditions, patient population, host factors, prior antimicrobial exposure, and the organisms unique to each facility may govern the similarity and or differences in the spectrum of pathogens implicated in causing catheter-associated bacteriuria and/or candiduria in the ICU patients [34].

The degree of antimicrobial resistance against both Gram-positive and gram-negative bacteria in our study was striking. Gram-negative bacteria demonstrated a high percentage of antibiotic resistance to almost all antibiotic categories tested with a relatively lower prevalence of resistance to amikacin 8(16.0%), nitrofurantoin 4(17.4%), meropenem 10 (20.0%), and imipenem 10(20.0%). The highest percentage of resistance against gram-negative bacteria was exhibited towards ceftriaxone 36(94.7%), ampicillin 21(91.3%), cefotaxime34(89.5%), and trimethoprim-sulfamethoxazole 34(81.6%). Contrary to our finding, however, Fahim [39] reported a comparable high percentage of antibiotic resistance towards nitrofurantoin (52.5%), amikacin (58.0%), imipenem (59.7%), and meropenem (62.0%) in Gram-negative bacteria recovered from ICU patients. The degree of antimicrobial resistance against Gram-positive bacteria was relatively low. Indiscriminate use of broad-spectrum antibiotics empirically since there is no culture facility in our ICU and lack of an antibiotic stewardship program may all have contributed to the high antibiotic resistance rates in this study.

The most worrisome finding in this study was that almost all the gram-positive and Gram-negative bacteria developed MDR strains at varying frequencies. Multi-antibiotic resistance has been identified as one of the ten threats and it has been increasing at a very high-rate worldwide [40] it is more common in healthcare-associated infections than community-acquired infections. In line with this, the percentage prevalence rate of MDR in Gram-negative bacteria, particularly in *Enterobacteriaceae* was extremely high. For example, all isolates of *E*. *coli* and *Klebsiella* species were multiantibiotic-resistant. The ubiquity of *Enterobacteriaceae* in our settings and animal hosts and the relative ease acquisition of plasmids containing genes that encode for Extended Spectrum β- Lactams (ESBLs) and other resistance genes that confer resistance to many other classes of antibiotics contributed to a high percentage prevalence rate of MDR in our current study [41–44]. The antimicrobial resistance patterns and the high percentage of MDR reported in our study towards non- fermentative Gram-negative bacteria such as species of *Pseudomonas and Acinetobacter* also demonstrated a series of concerns of this group of Gram-negative bacteria in CAUTI in ICU patients. The overall percentage prevalence of MDR against gram-positive bacteria was lower than Gram-negative bacteria (28.9 vs 55.6%) in which most of them were accounted for by *Enterococcus* strains.

## Conclusions

A very high prevalence of bacteriuria/ candiduria was demonstrated in this study. This warrants the establishment of a multidimensional infection control approach on catheter-associated urinary tract infection in ICU. In addition to a high prevalence rate of candiduria, recovery of non-albicans candida species almost in equal proportion with candida albicans in the present study was an important finding as non-albicans candida species are intrinsically resistant to the commonly used azole antifungal drugs in Ethiopia. The prevalence of rate MDR bacteria in our ICU particularly of E. coli, Klebsiella spp, Pseudomonas, and Acetobacter spp was very high. To combat this problem, proper antibiotic policies should be formulated.

## Supporting information

S1 Data Minimal study data. (SAV)
